# Supplementary material for: Precipitation of corrosion products in macroscopic voids at the steel–concrete interface: observations, mechanisms and research needs
Source: Mater Struct. 2025 Mar 13;58(3):90. doi: 10.1617/s11527-025-02614-z (PMC11906557; doi:10.1617/s11527-025-02614-z)
Supplement: Supplementary file 1 — (DOCX 6680 KB) [file 11527_2025_2614_MOESM1_ESM.docx]

**Supplementary Information: Precipitation of corrosion products in macroscopic voids at the steel–concrete interface: observations, mechanisms and research needs**

Shishir Mundra^1,a^, Emanuele Rossi^1^, Luka Malenica^1^, Mohit Pundir^1^, Ueli M. Angst^1^

^1^Institute for Building Materials, ETH Zürich, Laura-Hezner-Weg 7, 8093, Zürich, Switzerland

Corresponding author: ^a^ [smundra@ethz.ch](mailto:smundra@ethz.ch)

[1. Supplementary Note 1 2](#_Toc169537467)

[2. Supplementary Note 2 3](#_Toc169537468)

[3. Supplementary Note 3 7](#_Toc169537469)

1. **Supplementary Note 1**

*Methods: X-ray computed tomography*

We performed several X-ray Computed Tomography (XCT) tests of two cylindrical reinforced mortar specimens made with mixing proportions of 2:5 of Ordinary Portland cement (OPC) and aggregates (D_max_ = 2 mm), respectively, and a w/c of 0.45. In each specimen, the diameter of the mortar cylinder was 20 mm, in which a smooth carbon steel rebar with an external diameter of 6 mm is centrally embedded, resulting in a cover depth of 7 mm. Before casting, a wire is soldered to one edge of each steel rebar to enable electrochemical measurements of the steel reinforcement. Subsequently, both edges of each steel rebar are coated first with Teflon tape (25 mm in length at each edge), followed by heat-shrinkable membranes (20 mm in length at each edge) to minimize edge effects and prevent corrosion initiation in these sections. During mortar casting, the specimens were mechanically vibrated using a vibrating table for 30s. After casting, the samples undergo a 28-day curing process (T = 20 ± 1 °C, RH = 95 ± 1%). Following curing, the edges of the reinforced concrete specimens are sealed with Teflon and hydraulic tape, leaving a 30 mm-exposed central area, corresponding to the region under investigation. One initial acquisition of the specimens was performed in their dry state after 28 days of hydration at RH > 95% and 20 °C (Figure 3a-b and Figure 5a – main manuscript). After that, one of the two specimens (which we refer to in Figure 3) was exposed to wet-dry cycles with 3.5 wt.% NaCl solution with a duration of 8 and 64 hours, respectively. During the wet cycles, samples were submerged in 3.5 wt.% NaCl solution. XCT acquisitions were performed after 8 weeks (Figure 3b-c – main manuscript) and 12 weeks (Figure 3c-d – main manuscript) of wet-dry cycles, as well as after 4 weeks of prolonged drying (Figure 3e-f – main manuscript) under laboratory conditions at the end of the 12-weeks-long exposure to corrosive conditions. It is to be noted that all XCT data were acquired during the drying phase of the wet-dry cycles.

The other specimen, to which we refer in Figure 5 (main manuscript), was kept submerged in 3.5 wt.% NaCl solution and imaged after 8 weeks (Figure 5b – main manuscript). The acquisition was conducted after taking the sample out of the exposure solution and wrapping it with parafilm just before each XCT acquisition. All the XCT acquisitions were conducted at the ICON beamline of Paul Scherrer Institut (PSI, Villigen, Switzerland) [1], with an acceleration voltage of 150 keV to acquire 1125 projections over 360° of rotation, each one with an exposure time of 20 seconds. The reconstruction of the XCT acquisitions was performed using MuhRec [2]. The outcome of each reconstruction was a stack of 2175 images with 1791 x 1791 pixels (16 bits) per image, with a final voxel size of 13.8 µm.

1. **Supplementary Note 2**

*Numerical methods: Computation fluid dynamics simulations*

In this work, an OpenFOAM-based Volume-of-Fluid (VOF) solver [3] is used to model air-water multiphase flow directly at the pore level [4]. Air-water multiphase flow at the pore scale is governed by a one-fluid formulation of the incompressible Navier-Stokes equations consisting of mass and momentum conservation [5] (Eq. S1 and Eq. S2):

∇ ⋅ 𝐮 = 0 (Eq. S1)

$\frac{\partial\rho\mathbf{u}}{\partial t}+\nabla\cdot(\rho\mathbf{uu}) = -\nabla p+\nabla\cdot\{\mu[\nabla\mathbf{u}+{(\nabla\mathbf{u})}^{T} ]\}+\rho\mathbf{g}+\mathbf{f}_{\mathrm{st}}$ (Eq. S2)

where **u** is velocity vector, *t* is time, *p* is pressure, *ρ* is density, *μ* is viscosity, **g** is gravity vector and **f_st_** is surface tension force. For the case where surface tension is constant, the term **f_st_** can be modelled by the Continuous Surface Force (CSF) model, as shown in Eq. S3 [6]:

$\mathbf{f}_{\mathrm{st}}$ = 𝜎𝜅𝐧$\delta_{\Gamma}$ (Eq. S3)

Where, *σ* is the surface tension, κ is the curvature of the interface, **n** is the normal vector to the fluid/fluid interface, and *δ* is the Dirac delta function located at the interface Γ.

Eq. S1 and S2 are valid even in the case of multiphase flow, where fluid properties (density and viscosity) change discontinuously across the air-water interface. However, the movement of the interfaces (i.e., the position of different phases) needs to be modelled, and there are different computational approaches to track the interface.

We use the VOF approach in this work [7]. In this approach, the interface is tracked using an indicator function *α*, representing the volume fraction of one of the fluids in each computational cell. It takes value *α* = 1 for the first fluid and *α* = 0 for the second fluid, while the interface is located at the position where fluid properties change abruptly. All flow variables in the VOF method are defined using their single-filed values (Eq. S4 – S7):

$\rho= \alpha\rho_{w}+ (1 - \alpha)\rho_{a}$ (Eq. S4)

$\mu= \alpha\mu_{w}+ \left( 1 - \alpha\right)\mu_{a}$ (Eq. S5)

$\mathbf{u} = \alpha\mathbf{u}_{w}+ \left( 1 - \alpha\right)\mathbf{u}_{a}$ (Eq. S6)

$p = \alpha p_{w}+ (1 - \alpha)p_{a}$ (Eq. S7)

where, subscripts *w* and *a* denote the water and air phase, respectively. The movement of the interface is obtained by solving an additional advection equation (Eq. S8):

$\frac{\partial\alpha}{\partial t}+\nabla\cdot(\alpha\mathbf{u}) = 0$ (Eq. S8)

while, the normal vector (**n**) and curvature (κ) (required to calculate surface tension term in Eq. S3) are calculated through Eq. S9 and Eq. S10:

$\mathbf{n}=\frac{\nabla\alpha}{||\nabla\alpha||}$ (Eq. S9)

$\kappa=\nabla\cdot\mathbf{n}=\nabla\cdot\left( \frac{\nabla\alpha}{||\nabla\alpha||} \right)$ (Eq. S10)

The contact angle *θ*, representing the angle where the air-water interface meets the solid surface, defines the surface wettability properties. In the VOF method, the contact angle is defined as a boundary condition and incorporated by modifying a normal vector at the solid surface by the following relation (Eq. S11):

${\mathbf{n}\mathbf{=}\mathbf{n}}_{s}\cos\theta+\mathbf{t}_{s}\sin\theta$ (Eq. S11)

where, **n_s_** and **t_s_** are the normal and tangent unit vectors, respectively.

In all examples, we use the following air and water properties: *ρ_w_* = 1000 kg/m^3^, *ρ_a_* = 1.225 kg/m^3^, *μ_w_* = 10^−3^ Pa⋅s, *μ_a_* = 1.81 × 10^−5^ Pa⋅s and *σ* = 0.072 N/m. The contact angle boundary conditions are set to *θ*_cement_ = 0° and *θ*_steel_ = 75 ° for cement and steel surfaces, respectively. Gravitational acceleration *g* (m/s^2^) is neglected in this work as 2D representation is assumed to be in the horizontal plane. Anyway, the gravity effects on the flow dynamics at the pore scale are often negligible (as the capillary forces are dominating) until the air gets trapped. Once the air bubble is trapped, the gravity effects could play a role and its influence will depend on the size of the trapped bubble. While larger bubbles are expected to rise due to buoyancy, the movement of smaller bubbles can be affected by Brownian motion.

*How does water ingress in a macroscopic void at the SCI?*

Figure S 1 shows a schematic of the simulated water-filling process of a macroscopic void at the SCI, surrounded by a simplified capillary system, where the macroscopic void is initially filled with incompressible air. The air-liquid interface has a concave meniscus (Figure S 1a) indicating that capillary forces (driving force resulting from large capillary pressure due to the small diameter of the inlet capillary) dominate over imposed external pressure difference. Once Pore A is filled, the ingress of water significantly slows down due to a lower driving force resulting from the larger curvature of the air-liquid interface in the large void (Figure S 1b-d). However, dominant viscous dissipation is still happening inside Pore A due to the flow of a more viscous fluid (water, when compared to air) between its narrow walls (i.e., friction effects). Once water enters the void, the meniscus eventually reaches the outlet capillaries (Pore B and C) as shown in Figure S 1c. The junctions between the void and the outlet capillaries represent geometrical barriers for the liquid meniscus to advance along the walls of the void and, as a result, the air-liquid interface is locally pinned and will not further (at least momentarily) advance along the void walls. Because of this, the pressure in the liquid phase starts to build-up leading to changes in the curvature of the air-liquid interface (compare meniscus curvature between Figure S 1c and Figure S 1d) and the shape of the meniscus eventually changes from concave to convex (Figure S 1e). This indicates that the pressure in the liquid phase changed from negative (under-pressure) to positive (over-pressure). Thus, the capillary pressure now acts as an opposing force for further imbibition of water, however, ingress of liquid (and its consequent slow progress into the void) continues due to the presence of an external pressure difference.





Figure S 1: Schematic of the simulated water-filling process of a coarse void (Ø 1 mm) at the SCI over time (a to e). The interfacial void is connected to one inlet capillary of 40 µm (Pore A) and two outlet capillaries of 45 µm and 25 µm (Pores B and C, respectively), through which air can flow while being replaced by the liquid. All other boundaries are considered impermeable surfaces with a predefined contact angle (𝜃) of 75° at the steel surface and 0° degrees at the walls of the porous network (both void and capillaries). Furthermore, the initial and outlet pressures are set to zero, while pressure at the inlet is kept to 0.1 bar. The interfacial void is initially dry (Figure S 1a), and water ingress resembles the flow through the concrete cover, where the inlet capillary (Pore A) has a contraction to simulate viscous dissipation in the concrete cover.

*Influence of surrounding capillary pores*

To gain insight on the influence of capillary interconnectivity on water ingress into the void, we use a simple scenario where we consider the inlet and outlets pores interconnected by two additional capillary pores with a size of 20 µm (AB) and 25 (AC) μm, as visible in Figure S 2.





Figure S 2:Water filling process of a macroscopic void at the SCI surrounded by a simplified interconnected capillary pore network. 𝜃 = 0° at the edges of the porous network and 𝜃 = 75° at the steel surface.

Figure S 2 clearly shows that the interconnectivity of the capillary porous network drastically influences the void filling process (and thus, the actual air-water distribution within the macroscopic void). Since capillary suction is inversely proportional to the radius of the pore in which it occurs, the transport of water (and saturation) in capillary pores is significantly quicker than that in macroscopic voids [8,9]. Water ingress into the macroscopic void is only marginal since the saturation of the capillary porosity surrounding the void does not provide any pathway for the gaseous phases to escape the void, resulting in the entrapment of the gas phase which prevents further water ingress. Under these conditions, the complete saturation of macroscopic voids would involve the dissolution of the entrapped air [8,9] which, theoretically, may take decades to occur. However, partially filled interfacial coarse voids with a significantly higher degree of saturation at significantly shorter durations of exposure (8 weeks) is observed from our XCT results shown in Figure 5b (main manuscript). Therefore, characterisation (at the nm scale) of the capillary pore network around the macroscopic interfacial void is essential to better understand water ingress in macroscopic voids.

1. **Supplementary Note 3**

We consider the growth of a corrosion product solely due to the differences in the relative densities of the Fe(III) complex in its aqueous form and in its solid form. For example, Fe(III) in aqueous solutions has a density equal to that of water, and when precipitating as goethite (solid corrosion product), has a density ten times that of water. Furthermore, an aqueous Fe(III) complex transitions into a solid form under supersaturation conditions i.e. when the concentration is above the solubility limit of the precipitating solid corrosion product. The molar volume of the precipitating corrosion products is much higher than that of their aqueous form. As a result, during the process of precipitation, a precipitate would want to achieve the volume corresponding to its density (*V_ppt_*). Therefore, for every mol, they occupy more volume than their aqueous counterpart (*V*). This change in the volume *ΔV* (*= V_ppt_ - V*) is referred as the expansion of the precipitate layer (see Figure S3a). In the absence of any physical restrictions, the precipitate layer would reach this volume (*V_ppt_*). However, when it expands under certain constraints, such as the surrounding medium, it expands to a volume that is smaller than the expected one, resulting in stress within the precipitate layer and the surrounding medium. The stress-induced within the precipitated layer thus can be expressed as (Eq. S12):

$\sigma_{\mathrm{ppt}}=\lambda\cdot tr(\varepsilon- \Delta V/V)+2\mu(\varepsilon- \Delta V/V)$ (Eq. S12)

where, *ε* is the total strain in the precipitate layer due to the volume expansion and due to the internal elastic stress (*ε = ΔV/V + ε*_elastic_). *λ* and *μ* are the Lames' parameters of the corresponding materials. Similarly, for the surrounding media (without the volume expansion), the stress-strain relationship is given as (Eq. S13):

$\sigma_{\mathrm{cem}}=\lambda\cdot tr(\varepsilon_{\mathrm{elastic}})+2\mu(\varepsilon_{\mathrm{elastic}})$ (Eq. S13)

We assume that the layer of precipitated corrosion products is perfectly attached to the surrounding media at the walls of the macroscopic interfacial void *i.e.*, there is no slip or detachment along the void-cement interface. Thus, the governing equation (under no body force or applied traction) for the two phases is given as (Eq. S14):

$\sum_{i} \nabla\cdot\sigma_{i}=0$ (Eq. S14)

where *i* indicates different phases (corrosion products, water, cementitious material). We consider three different cases at the SCI:

1. A single layer of corrosion products precipitating on the walls of a completely saturated macroscopic void *i.e.*, 3 phases in total: cement phase (outer), precipitate layer (middle), and water (inner),
2. Two layers of corrosion products precipitating within a completely saturated macroscopic void *i.e.*, 4 phases in total: cement phase (outer), 1^st^ layer of corrosion products, 2^nd^ layer of corrosion products and water (inner-most), and,
3. Corrosion products precipitating with the macroscopic void and expanding from inside to outside *i.e.*, only 2 phases: cement phase (outer) and precipitate (inner).

For all the considered cases, we assume that the displacements at the outermost boundary of the cementitious material are 0. The material properties (Youngs modulus: *E*, and Poisson’s ratio: *ν*) [10–12] considered for the different cases are as follows:

$E_{\mathrm{cem}}=10 GPa=10\times{10}^{9} N/m^{2}, \nu=0.2$

$E_{\mathrm{geothite}}=100 GPa=100\times{10}^{9} N/m^{2}, \nu=0.45$

$E_{\mathrm{magnetite}}=200 GPa=200\times{10}^{9} N/m^{2}, \nu=0.45$

$E_{\mathrm{water}}=2.2 MPa=2.2 \times{10}^{6} N/m^{2}, \nu=0.49$

We employ the Finite Element method to investigate the effect of a growing precipitate layer on the stress state within the layers and in the surrounding media. In the Figure S 3b, we show the normalised stress state for the case where a precipitate expands from inside the pore towards its boundary.


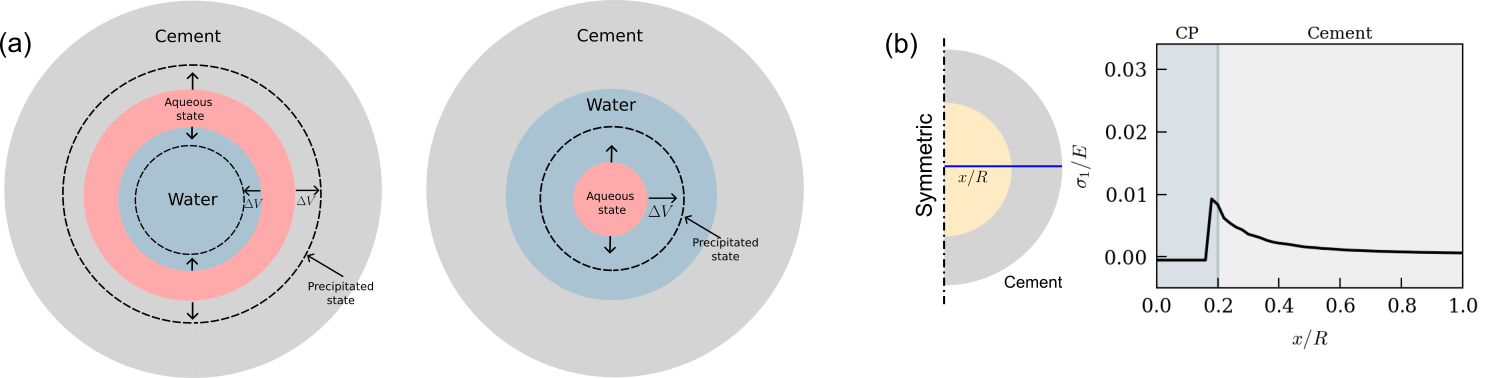


Figure S 3: (a) Schematic showing how the transition from aqueous Fe(III) to the solid corrosion products leads to expansion. Here, we show the cases when the precipitation of corrosion products (CP) occurs along the walls of the macroscopic void and when the precipitation happens at the center of the pore. Upon precipitation in the first case, the aqueous layer (that precipitates as solid corrosion products) along the walls of the void would want to expand in both directions, whereas in the second case, the aqueous layer (precipitating as a solid corrosion product) expands only outwards upon precipitation. Figure (b) shows the normalized major principal stresses inside the corrosion product layer and the surrounding cementitious phase for the case when the expansion happens from the inside to the outside.

1. **References**

[1] A.P. Kaestner, J. Hovind, P. Boillat, C. Muehlebach, C. Carminati, M. Zarebanadkouki, E.H. Lehmann, Bimodal Imaging at ICON Using Neutrons and X-rays, Phys Procedia 88 (2017) 314–321. https://doi.org/10.1016/j.phpro.2017.06.043.

[2] A.P. Kaestner, MuhRec - A new tomography reconstructor, Nucl Instrum Methods Phys Res A 651 (2011) 156–160. https://doi.org/10.1016/j.nima.2011.01.129.

[3] J. Maes, H.P. Menke, GeoChemFoam: Direct modelling of flow and heat transfer in micro-CT images of porous media, Heat and Mass Transfer/Waerme- Und Stoffuebertragung 58 (2022) 1937–1947. https://doi.org/10.1007/s00231-022-03221-2.

[4] L. Malenica, Z. Zhang, U. Angst, Towards improved understanding of spontaneous imbibition into dry porous media using pore-scale direct numerical simulations, Adv Water Resour 194 (2024) 104840. https://doi.org/10.1016/j.advwatres.2024.104840.

[5] G. Tryggvason, R. Scardovelli, S. Zaleski, Direct Numerical Simulations of Gas–Liquid Multiphase Flows, Cambridge University Press, Cambridge, 2011. https://doi.org/DOI: 10.1017/CBO9780511975264.

[6] J.U. Brackbill, D.B. Kothe, C. Zemach, A continuum method for modeling surface tension, J Comput Phys 100 (1992) 335–354. https://doi.org/10.1016/0021-9991(92)90240-Y.

[7] C.W. Hirt, B.D. Nichols, Volume of fluid (VOF) method for the dynamics of free boundaries, J Comput Phys 39 (1981) 201–225. https://doi.org/10.1016/0021-9991(81)90145-5.

[8] G. Fagerlund, A service life model for internal frost damage in concrete, Lund, Sweden, 2004.

[9] G. Fagerlund, Moisture design with regard to deterioration of materials and structures-with special reference to frost destruction, Lund, Sweden, 2006. http://lup.lub.lu.se/record/633721.

[10] E. Rossi, H. Zhang, S.J. Garcia, J. Bijleveld, T.G. Nijland, O. Çopuroğlu, R.B. Polder, B. Šavija, Analysis of naturally-generated corrosion products due to chlorides in 20-year old reinforced concrete: An elastic modulus-mineralogy characterization, Corros Sci 184 (2021) 109356. https://doi.org/10.1016/j.corsci.2021.109356.

[11] M. Pundir, D.S. Kammer, U. Angst, An FFT-based framework for predicting corrosion-driven damage in fractal porous media, J Mech Phys Solids 179 (2023) 105388. https://doi.org/10.1016/j.jmps.2023.105388.

[12] J.S. Jacobsen, P.N. Poulsen, J.F. Olesen, K. Krabbenhoft, Constitutive mixed mode model for cracks in concrete, Eng Fract Mech 99 (2013) 30–47. https://doi.org/10.1016/j.engfracmech.2013.01.004.
